# Sex differences in the diabetes-related risk of incident myocardial infarction and relationship to established coronary heart disease risk factors: the Tromsø Study (1979–2016)

**DOI:** 10.1093/ehjopen/oeag174

**Published:** 2026-10-21

**Authors:** Grethe Albrektsen, Tom Wilsgaard, Ivar Heuch, Dag S Thelle, Maja-Lisa Løchen, Sameline Grimsgaard, Kaare H Bønaa

**Affiliations:** Department of Public Health and Nursing, Faculty of Medicine and Health Sciences, NTNU Norwegian University of Science and Technology, P.O. Box 8905, 7491 Trondheim, Norway; Department of Community Medicine, Faculty of Health Science, UiT The Arctic University of Norway, P.O. Box 6050, 9037 Tromsø, Norway; Department of Mathematics, University of Bergen, P.O. Box 7803, 5020 Bergen, Norway; Department of Biostatistics, Institute of Basic Medical Sciences, University of Oslo, P.O. Box 1122, 0317 Oslo, Norway; School of Public Health and Community Medicine, Sahlgrenska Academy, Gothenburg University, P.O. Box 469, 405 30 Gothenburg, Sweden; Department of Clinical Medicine, UiT The Arctic University of Norway, P.O. Box 6050, 9037 Tromsø, Norway; Department of Cardiology, University Hospital of North Norway, P.O. Box 100, 9038 Tromsø, Norway; Department of Community Medicine, Faculty of Health Science, UiT The Arctic University of Norway, P.O. Box 6050, 9037 Tromsø, Norway; Heart and Thorax Clinic, St. Olavs University Hospital, P.O. Box 3250, 7006 Trondheim, Norway; Department of Circulation and Medical Imaging, Faculty of Medicine and Health Sciences, NTNU Norwegian University of Science and Technology, P.O. Box 8905, 7491 Trondheim, Norway

**Keywords:** Myocardial infarction, Diabetes, CHD risk factors, Sex differences

## Abstract

**Aims:**

To examine whether the adverse effect of diabetes on the risk of incident myocardial infarction (MI) differs between sexes and assess to what extent established coronary heart disease (CHD) risk factors contribute to the elevated risk.

**Methods and results:**

Prospective study of 42 907 Tromsø Study participants (51.5% women) with 3942 incident MI (357 with diabetes) at ages 35–104 years. Relative risks [incidence rate ratio (IRR)] were estimated in Poisson regression analyses. Impact of CHD risk factors was quantified as a percentage change in the regression coefficient for diabetes. A contrast in risk of MI by diabetes status was seen from age 45 years in men and 55 years in women. Adjusted for age and birth cohort, the IRR was 2.26 in women and 1.84 in men (*P* = 0.12, test for interaction). In a mutually adjusted model, adjustment for the triglyceride-to-HDL-cholesterol ratio weakened the association with diabetes similarly in women (−19.0%) and men (−17.0%); systolic blood pressure had a larger impact in women (−10.3% vs. +2.2%), whereas smoking strengthened the association more in men (+23.1% vs. +7.1%). Body mass index had a minor impact. A larger joint impact of adjustment factors in women (−33.6% vs. −15.9%) narrowed the initial gender gap (adjusted IRR of 1.72 and 1.67, respectively). Adverse lipid levels and increasing systolic blood pressure emerged already during the pre-diabetic phase.

**Conclusion:**

Traditional CHD risk factors contributed substantially to the diabetes-related risk of incident MI. A larger joint impact of these factors in women reduced the initial observed sex difference in the adverse effect of diabetes.

## Graphical Abstract

**Graphical Abstract:**
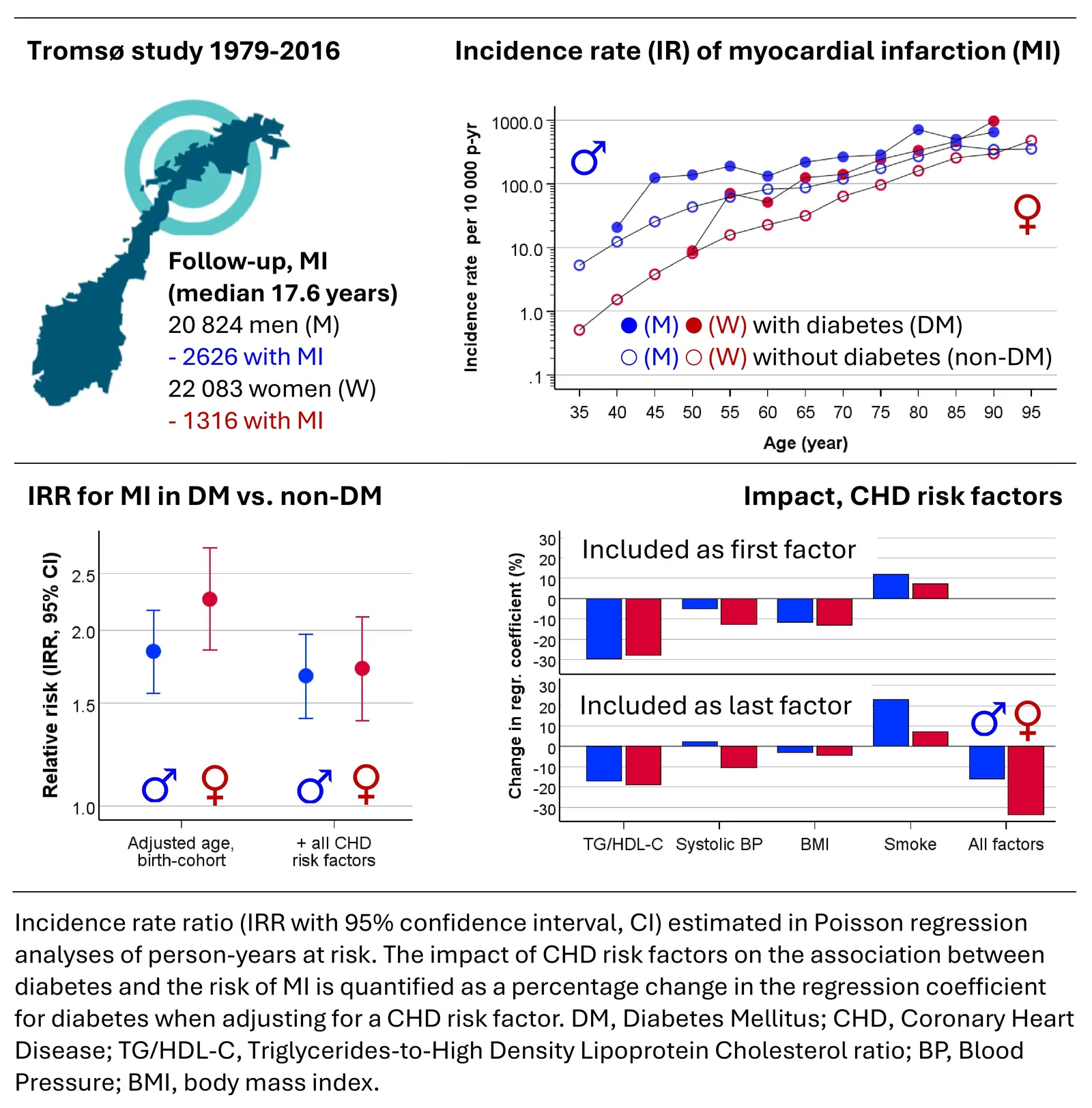

## Introduction

Diabetes is associated with an overall two- to four-fold increased risk of coronary heart disease (CHD) and the adverse effect has been found to be larger in women than in men.^1–5^ The excess risk in terms of absolute differences in risk appears to be rather similar in men and women, though with some variation across age groups.^6–10^ Despite numerous studies on associations between diabetes and the risk of CHD, few have quantified to what extent established CHD risk factors contribute to the elevated risk or whether the impact of these factors differs between sexes. Individuals with diabetes often have high systolic blood pressure and adverse lipid levels,^2,5^ especially high triglyceride levels in combination with low high-density lipoprotein cholesterol (HDL-C) levels.^11,12^ The contrast in the levels of these factors according to diabetes status has been found to be larger in women than in men,^13,14^ possibly leading to a larger impact on the association between diabetes and the risk of CHD in women compared to men. Moreover, the effect of blood pressure is found to be more pronounced in women than in men,^15,16^ and adverse triglyceride levels appear to be a stronger marker for subsequent incident myocardial infarction (MI) in women, especially after age 75 years.^17^ To our knowledge, no previous studies have examined whether sex differences in the effect of adjustment factors contribute to the observed sex difference in the adverse effect of diabetes. Thus, it is still not clear whether the adverse effect of diabetes in relation to the risk of CHD is larger in women than in men.

The aim of the present study was to examine whether the adverse effect of diabetes on the risk of MI differs between men and women and assess to what extent established CHD risk factors, including potential sex differences in their effects, contribute to the diabetes-related elevated risk. Heterogeneity by age is also examined. Results from our study are based on repeated measurements of CHD risk factors and self-reported diabetes from large population-based, health surveys conducted in Tromsø, Norway, linked to diagnosis of MI from local and national disease registers.

## Methods

### Study population

The present population-based study comprised 42 907 individuals (51.5% women) who participated in at least one of the six health surveys conducted in Tromsø, Norway, during the period 1979–2016 (the second to seventh Tromsø Study), were at least 20 years old at study participation and at least 35 years at the closing date of follow-up (31 December, 2019). The Tromsø Study was initiated in 1974 and the primary aim was to determine causes of the high cardiovascular mortality in Northern Norway (https://uit.no/research/tromsostudy). Data are assessed from population-based health surveys conducted at regular time intervals (6–7 years apart) in the municipality of Tromsø, Norway. About 45 000 inhabitants have participated in at least one survey so far. The first survey included only men (8867 individuals aged 20–49 years invited, attendance rate 74.4%), and data from this survey was not utilized in the present study. The attendance rate was high also for subsequent surveys (65–77%), but the number and age range of participants (new and previous) varied (in the range of 8130 to 27 158 participants; mainly aged ≥20 or ≥30 years). A total of 39.3%, 19.8%, 14.9%, 12.3%, 8.7%, and 4.9% of the individuals in our study cohort had participated in one, two, three, four, five and six surveys, respectively.

### Assessment of CHD risk factors

At each health survey, participants completed a questionnaire regarding demographic and clinical characteristics, lifestyle factors and health, including a question on whether they had diabetes. At fourth and later surveys, age at onset of diabetes was also assessed. Age at the survey participation when first reporting having diabetes was used as a substitute if age at diabetes onset was not explicitly reported. Leisure time physical activity, as an average of summer and winter periods, was reported as low (sedentary activity only, like TV-watching), moderate (walking/gardening at least 4 h per week), high (recreational sport activities at least 4 h per week) and very high (hard training several times a week). Anthropometric measures (including height and weight), blood pressure and blood samples (non-fasting) were assessed through physical examinations. Further details on the procedures applied, including assessment of serum lipid variables, are given elsewhere.^18,19^ Body mass index (BMI) was calculated in the present study as weight (in kilogram) divided by height (in metre) squared. In the classification of underweight, normal weight, overweight, obese I, and obese II, cutpoints at 18.5, 25, 30, and 35 were applied. Five clinically relevant categories were defined for pulse pressure, the difference between systolic and diastolic blood pressure (<40, 40–49, 50–59, 60–69, ≥70 mmHg).

### Assessment of end points

Data from the Tromsø Study were retrospectively linked to dates of diagnosis of MI, emigration or death by means of the unique Norwegian 11-digit personal identification numbers. Dates of diagnoses of MI (fatal and non-fatal) were assessed from the Tromsø Study Cardiovascular Disease Register (1979–2014, based on diagnoses from local hospital and discharge registers) and from the Norwegian Myocardial Infarction Registry (mandatory reporting) and the National Cause of Death Registry (2015–2019). The ICD codes applied to define MI are described elsewhere.^17^ Dates of emigration and death were obtained from the Central Population Register of Norway.

### Statistical analyses

Crude incidence rate (IR) of MI, as an estimate of absolute risk, for men and women with and without diabetes, was calculated as the number of MI cases divided by person-years at risk in 5-year age intervals during follow-up (see further details in Supplementary material online, Statistical analyses). Initially, each individual was considered at risk of MI from their first health survey participation at ages ≥20 years, until date of diagnosis of MI, emigration, death or closing date of study (31 December 2019). Since none of the women with diabetes, and few women without diabetes, was diagnosed with MI before age 35 years, the analyses were restricted to ages ≥35 years.

Incidence rate ratios of MI (IRR with 95% confidence intervals, CI) for individuals with vs. without diabetes were calculated as an estimate of relative risk in Poisson regression analyses of person-years at risk with age as time scale. The Poisson regression model is rather similar to the traditional Cox proportional hazard regression model except that the time scale is included as a parameter in the model (a full-parametric model). An interaction term between sex and an indicator variable for diabetes status (no, yes) at date of survey participation (corresponding in time with measurement of CHD risk factors) was included in the model to allow for sex differences in the adverse effect of diabetes. All the regression analyses were adjusted for age (in 1-year intervals, modelled as fourth-order polynomial) and birth cohort (5-year categories). Additional adjustment was made for a single or a set of traditional CHD risk factors. Serum lipid and blood pressure variables were treated as continuous covariates (modelled as linear trends), whereas pulse pressure (five 10 mmHg categories), BMI category (underweight/normal, overweight, obese I, obese II), daily smoking (no/previous, yes) and self-reported leisure time physical activity (low, moderate, high/very high) were treated as categorical covariates in the regression model. The impact of each single or set of CHD risk factor(s) on the association between diabetes and the risk of MI was quantified as a percentage change in the sex-specific regression coefficient for diabetes when included as an adjustment factor in the regression model. The impact of potential sex differences in the effect of an adjustment factor was evaluated by including an additional interaction term with sex. The regression analyses were restricted to 42 479 individuals aged 35–94 years with complete information on diabetes and CHD risk factors in the Tromsø Study. Additional 132 individuals were excluded due to missing value when adjusting for physical activity, but this factor was not considered in the final, mutually adjusted analyses.

The tabulation of individual data records into person-years tables with the number of MI cases and persons-years at risk in different risk factor categories during follow-up (data on aggregated group level) and the calculation of maximum likelihood estimates and two-sided likelihood ratio tests in the Poisson regression analyses were performed by means of the EPICURE program.^20^ The repeated measurements of CHD risk factors during follow-up were utilized in the generation of the person-years at risk table (see Supplementary material online, Statistical analyses).

### Additional analyses

The interpretation of relative risk estimates as a measure of excess risk depends on the risk level in the reference group.^1,21^ To account for the lower absolute risk of MI in women, additional analyses of a single variable representing combined categories of sex and diabetes status (a reparameterization of the ordinary interaction model) were carried out. This analytic approach makes it easier to compare the sex-specific IRR values since all subgroups are compared to the same reference group. Similarly, to account for the considerably lower risk in the youngest age group, an analysis of a single variable representing combined categories of age (35–54, 55–74, 75–94 years), sex and diabetes status was also performed. A previously developed analytic approach was utilized to avoid residual confounding by age when comparing IRR values across three broad age groups.^22^

To gain more insight into the role of the CHD risk factors adjusted for in the regression analyses, we examined temporal changes in BMI, blood pressure and lipid variables from time periods before to after diabetes onset (3081 measurements before, 2729 measurements after diabetes onset). Potential contrasts according to MI status at the end of follow-up were also explored.

Finally, to account for a potential diagnostic delay in diabetes onset, the Poisson regression analyses were repeated with the time-dependent indicator variable for diabetes reclassified according to a 10-year backdating. A total of 74 of the MI cases with diabetes onset within 10 years after being diagnosed with MI (50 men, 24 women) were then reclassified and contributed person-years at risk in the diabetes group in the last part of follow-up before MI.

## Results

### CHD risk factor levels at start and during follow-up

A total of 1857 individuals (923 men, 934 women) in our cohort reported having diabetes at the start or during follow-up. The mean (or median) lipid levels, BMI and blood pressure according to age at survey participation in men and women with and without diabetes are shown in Supplementary material online, *Figures S1*–*S3*. Individuals reporting having diabetes had in general less favourable levels, except for a lower total cholesterol level. They were also less often smokers (19.7% vs. 35.1% in men, 20.1% vs. 33.4% in women) but more often previous smokers, a pattern more evident for men (51.0% vs. 33.0%) than women (35.4% vs. 28.3%). A larger proportion of the individuals with than without diabetes reported being physically inactive (27.2% vs. 18.6% of the men, and 30.6% vs. 19.1% of the women reported sedentary activities only).

### Absolute risk of MI by sex and diabetes status

During a median follow-up of 17.6 years (range 1 month to 33 years) at ages 35–104 years, a total of 3942 individuals (2626 men, 1316 women) were diagnosed with incident MI, wherein 357 cases (217 men, 140 women) were among individuals with diabetes. The number of MI cases and person-years at risk according to sex and diabetes status in 5-year age intervals during follow-up are shown in *Table 1*. The incidence rate of MI was higher in women with than without diabetes from ages around 55 years (*Figure 1*). Corresponding age for men was 45 years. The risk of MI in women with diabetes was rather similar to the risk in men without diabetes. However, men with diabetes had the highest risk during almost their entire life span (*Figure 1*).

**Figure 1:**
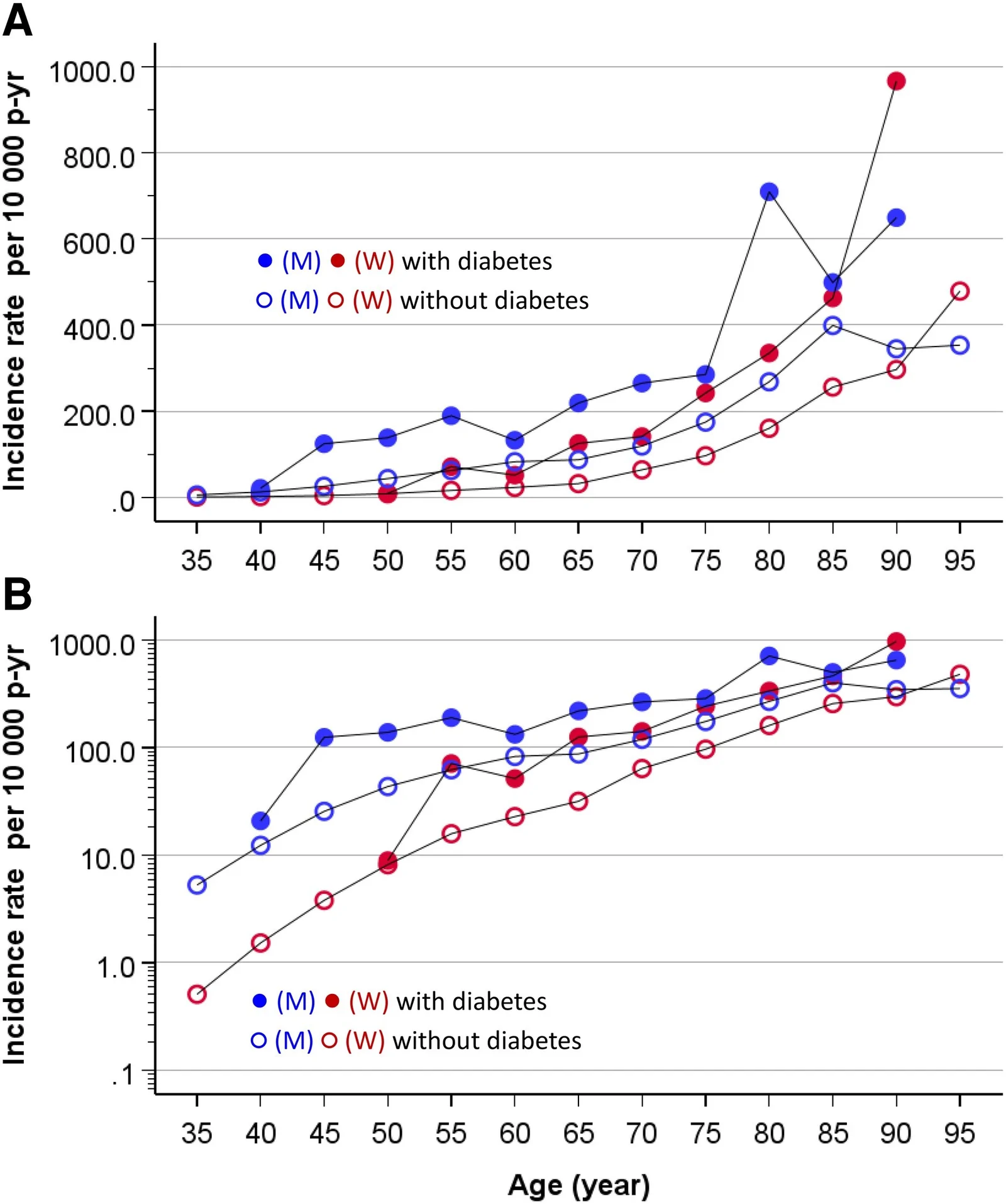
Crude age-incidence rate curves of myocardial infarction in men and women with and without diabetes, based on data from the Tromsø Study 1979–2016. The incidence rates (in 5-year age groups) are shown on (*A*) absolute scale and (*B*) logarithmic scale. P-yr, person-years; M, men; W, women.

**Table 1:** Crude age incidence rates of myocardial infarction in men and women with and without diabetes

	With diabetes (Dia)			Without diabetes (N-Dia)			Dia vs. N-Dia	
Age (year)	MI	P-yr × 10^4^	IR	MI	P-yr × 10^4^	IR	RD	IRR
**Men**								
35–39	1	307.8		28	53 251.0	5.3	–	–
40–44	1	656.8	20.7	82	66 692.6	12.3	8.4	1.7
45–49	13	1046.3	124.3	188	73 838.9	25.5	98.8	4.9
50–54	18	1302.5	138.2	302	69 747.7	43.3	94.9	3.2
55–59	29	1532.2	189.3	367	59 163.6	62.0	127.2	3.1
60–64	23	1738.1	132.3	377	45 716.4	82.5	49.9	1.6
65–69	36	1645.6	218.8	277	31 832.7	87.0	131.8	2.5
70–74	32	1207.9	264.9	245	20 601.3	118.9	146.0	2.2
75–79	23	806.6	285.1	217	12 435.7	174.5	110.6	1.6
80–84	30	422.8	709.6	181	6751.3	268.1	441.5	2.6
85–89	8	160.4	498.8	114	2857.8	398.9	99.8	1.3
90–94	3	39.8	649.4	27	783.2	344.7	304.7	1.9
≥95	0	6.4	–	4	113.3	353.2	–	–
**Women**								
35–39	1	411.0	–	3	59 201.9	0.5	–	–
40–44	0	751.5	–	11	72 341.0	1.5	–	–
45–49	0	1059.4	–	30	79 230.9	3.8	–	–
50–54	2	1157.5	8.8	59	72 599.4	8.1	0.8	1.1
55–59	8	1128.2	70.9	94	59 604.4	15.8	55.1	4.5
60–64	7	1363.2	51.4	105	46 180.4	22.7	28.6	2.3
65–69	18	1440.1	125.0	105	33 232.6	31.6	93.4	4.0
70–74	19	1348.5	140.9	154	24 185.8	63.7	77.2	2.2
75–79	27	1115.2	242.1	162	16 804.8	96.4	145.7	2.5
80–84	27	806.3	334.8	182	11 364.6	160.1	174.7	2.1
85–89	19	410.5	462.8	165	6448.4	255.9	206.9	1.8
90–94	11	118.5	966.7	76	2562.4	296.6	670.1	3.0
≥95	1	5.7	–	30	626.6	478.8	–	–

The Tromsø Study 1979–2016, with follow-up until 2020.

The age incidence rates per 10 000 person-years are calculated as the number of incident MI divided by person-years at risk within each 5-year age group, multiplied by 10^4^. Due to low numbers, the two (or four) youngest and the two oldest age groups, respectively, are combined in the subgroups with diabetes.

MI, myocardial infarction; IR, incidence rate; IRR, incidence rate ratio; P-yr, person-years; RD, risk difference.

### Impact of CHD risk factors on the diabetes-related elevated risk of MI

Among individuals aged 35–94 years with complete information on the CHD risk factors considered, the estimated overall adverse effect of diabetes in terms of relative risk was more pronounced in women (IRR = 2.26, 95% CI = 1.85–2.77) than in men (IRR = 1.84, 95% CI = 1.56–2.16) in the analyses adjusted for age and birth cohort (*Table 2*). This sex difference did not reach statistical significance (*P* = 0.12, test for interaction), but the 95% confidence intervals were wide. When including CHD risk factors one at a time in the model (*Table 2*), adjustment for triglyceride and HDL-C levels, and especially the ratio between these lipid variables, reduced the regression coefficient for diabetes the most and to a similar extent in both sexes. Adjustment for systolic blood pressure weakened the association with diabetes more in women than men, whereas adjustment for total cholesterol level and smoking strengthened rather than reduced the association with diabetes, with a larger impact in men than in women. Adjustment for BMI and pulse pressure weakened the association between diabetes and the risk of MI rather similarly in both sexes. Adjustment for physical activity had a minor impact (≤4%) and was not considered further, also due to the larger number of missing values.

**Table 2:** Incidence rate ratio (IRR with 95% CI) of myocardial infarction in individuals with vs. without diabetes

	Men	Women	Men	Women
	IRR (95% CI)	IRR (95% CI)	Δ%	Δ%
Adjusted age and birth cohort	**1.84** (**1.56–2.16)**	**2.26** (**1.85–2.77)**	***Ref.***	***Ref.***
**Additional adjustment**				
**Lipid variables**				
Total cholesterol (TC)	2.06 (1.75–2.42)	2.46 (2.01–3.02)	+18.8	+10.6
HDL-C	1.64 (1.39–1.93)	1.91 (1.55–2.34)	−18.6	−21.0
Triglycerides (TG)	1.60 (1.36–1.89)	1.90 (1.55–2.33)	−22.1	−21.1
HDL-C/TC (ratio)	1.73 (1.47–2.04)	1.95 (1.59–2.88)	−9.3	−18.3
TG/HDL-C (ratio)	1.53 (1.30–1.81)	1.80 (1.47–2.21)	−29.6	−28.0
**Blood pressure variables**				
Systolic BP	1.78 (1.51–2.10)	2.04 (1.66–2.50)	−5.1	−12.7
Diastolic BP	1.91 (1.62–2.25)	2.25 (1.84–2.76)	+6.5	−0.4
Pulse pressure	1.74 (1.48–2.05)	2.10 (1.72–2.58)	−8.5	−8.8
**Other factors**				
Body mass index	1.71 (1.45–2.01)	2.03 (1.65–2.49)	−11.8	−13.0
Daily smoking	2.01 (1.71–2.37)	2.40 (1.96–2.94)	+11.0	+7.4
Physical activity	1.81 (1.54–2.15)	2.25 (1.82–2.77)	−4.0	−3.4

Impact of adjustment for a single CHD risk factor (partly adjusted models).

Results from Poisson regression analyses of person-years at risk. The sex-specific IRR values are calculated on basis of a model including an interaction between sex and diabetes status. The impact of each CHD risk factor is quantified as percentage change (Δ%) in the regression coefficient for diabetes when including a CHD risk factor in a model adjusted for age and birth cohort (reference model).

BP, blood pressure; CHD, coronary heart disease; CI, confidence interval; HDL-C, high-density lipoprotein cholesterol.

In mutually adjusted analyses, blood pressure was represented by systolic blood pressure and serum lipid levels by HDL-C and/or triglyceride levels since these factors had a larger impact than the other lipid and blood pressure variables. The joint impact of all adjustment factors in the fully adjusted analyses was considerably larger in women than in men (*Table 3*, Supplementary material online, *Table S1*), narrowing the gender gap seen in the partly adjusted analyses (*Table 2*). The change in the regression coefficient for diabetes was in the range −28.5% to −35.4% in women (IRRs in range 1.69 to 1.79) and −9.9% to −15.9% in men (IRRs in range 1.68 to 1.73), depending on the model applied. The triglyceride–to-HDL-C ratio reduced the regression coefficient for diabetes the most also in the fully adjusted analyses (*Table 3*, Model 1), although the impact was weaker than in the partly adjusted model (a change from −29.6% to −17.1% in men and from −28.0% to −19.0% in women). Notably, the impact of triglycerides and HDL-C was considerably lower in a mutually adjusted model (*Table 3*, Model 2) than in the models including one factor at a time (see Supplementary material online, *Table S1*, Models 3 and 4).

**Table 3:** Incidence rate ratio (IRR with 95% CI) of myocardial infarction in individuals with vs. without diabetes

	Men	Women	Men	Women
	IRR (95% CI)	IRR (95% CI)	Δ%	Δ%
Adjusted age and birth cohort	**1.84 (1.56–2.16)**	**2.26 (1.85–2.77)**	***Ref.***	***Ref.***
**Additional adjustment**				
**Model 1**				
Joint impact, all factors	**1.67 (1.41–1.97)**	**1.72 (1.40–2.11)**	**−15.9**	**−33.6**
**Impact, each single factor**				
Triglycerides/HDL-C (ratio)	−17.1%	−19.0%		
Systolic BP	+2.2%	−10.3%		
Body mass index	−2.9%	−4.4%		
Daily smoking	+23.1%	+7.1%		
**Additional interaction term**				
With systolic BP	**1.71 (1.45–2.02)**	**1.63 (1.33–2.00)**		
	+5.3%	−9.9%		
**Model 2**				
Joint impact, all factors	**1.68 (1.42–1.98)**	**1.69 (1.38–2.08)**	**−14.7**	**−35.4**
**Impact, each single factor**				
Triglycerides	−5.5%	−6.5%		
HDL-C	−5.2%	−9.6%		
Systolic BP	+2.0%	−10.9%		
Body mass index	−2.8%	−3.9%		
Daily smoking	+22.7%	+7.6%		
**Additional interaction term**				
With systolic BP	**1.77 (1.50–2.09)**	**1.70 (1.38–2.08)**		
	+5.0%	−9.4%		

Impact of adjustment for CHD risk factors (mutually adjusted models).

Results from Poisson regression analyses of person-years at risk. The sex-specific IRR values are calculated on basis of a model including an interaction between sex and diabetes status. The joint impact of CHD risk factors is quantified as percentage change (Δ%) in the regression coefficient for diabetes when including all factors simultaneously in a model adjusted for age and birth cohort (reference model). The impact of a single CHD risk factor in a mutually adjusted model is calculated as percentage change when including this factor in the last step. The impact of including an additional interaction term to allow for sex differences in the effect of systolic blood pressure (*P* < 0.001, test for interaction) is also shown.

BP, blood pressure; CHD, coronary heart disease; CI, confidence interval; HDL-C, high-density lipoprotein cholesterol.

The sex difference in the impact of systolic blood pressure and smoking also remained in the mutually adjusted analyses (*Table 3*, Supplementary material online, *Table S1*). In men, the impact of smoking became stronger (increased from +11.0 to +20.1–23.1%, depending on how the lipid variables were included in the model) and had an even larger impact than adjustment for the triglyceride-to-HDL-C ratio, though in the opposite direction. Notably, the impact of BMI was considerably reduced and became negligible (<5%) after adjustment for the triglyceride-to-HDL-C ratio (*Table 3*, Model 1).

The regression coefficient for diabetes in women was further reduced (9–10%) when including an additional interaction term with sex to account of a significantly stronger effect of systolic blood pressure in women compared to men (*P* < 0.001, test for interaction; *Table 3* and Supplementary material online, *Table S1*). Adding an interaction term between sex and smoking status (*P*, test for interaction in range <0.001 to 0.16) or between sex and total cholesterol (*P* < 0.001, test for interaction in a model with lipid level represented by total cholesterol) had no additional influence. The effect of other adjustment factors did not differ significantly by sex.

### Age-specific analyses

Age-specific analyses (<55, 55–74, and 75–94 years, respectively) indicated a stronger adverse effect of diabetes on the risk of MI in the youngest men (see Supplementary material online, *Table S2*). The contrast in risk according to diabetic status decreased with increasing age in men. In women, no adverse effect of diabetes was seen before age 55 years despite similar amounts of person-years at risk to men (*Table 1*). The IRR values in middle-aged and older women were rather similar (see Supplementary material online, *Table S2*).

In all age groups, the triglyceride-to-HDL-C ratio remained the factor that weakened the association between diabetes and the risk of MI the most (see Supplementary material online, *Table S2*). Among the oldest men (≥75 years), adjustment for this lipid ratio had an even larger impact than adjustment for smoking. In women, adjustment for systolic blood pressure and the triglyceride-to-HDL-C ratio had rather similar impact in middle-aged (55–74 years), whereas the triglyceride-to-HDL-C ratio had the largest impact at ages ≥75 years.

### Analyses of combined categories of sex, age, and diabetes status

Relative risk estimates from the additional analyses with a common reference group confirmed that the diabetes-related elevated risk of MI is just as large in men as in women after adjustment for established CHD risk factors (*Figure 2A*). These supplementary analyses also indicated a more pronounced adverse effect of diabetes in young compared to older men, as well as a more pronounced adverse effect of diabetes in men than women before age 55 years (*Figure 2B*).

**Figure 2:**
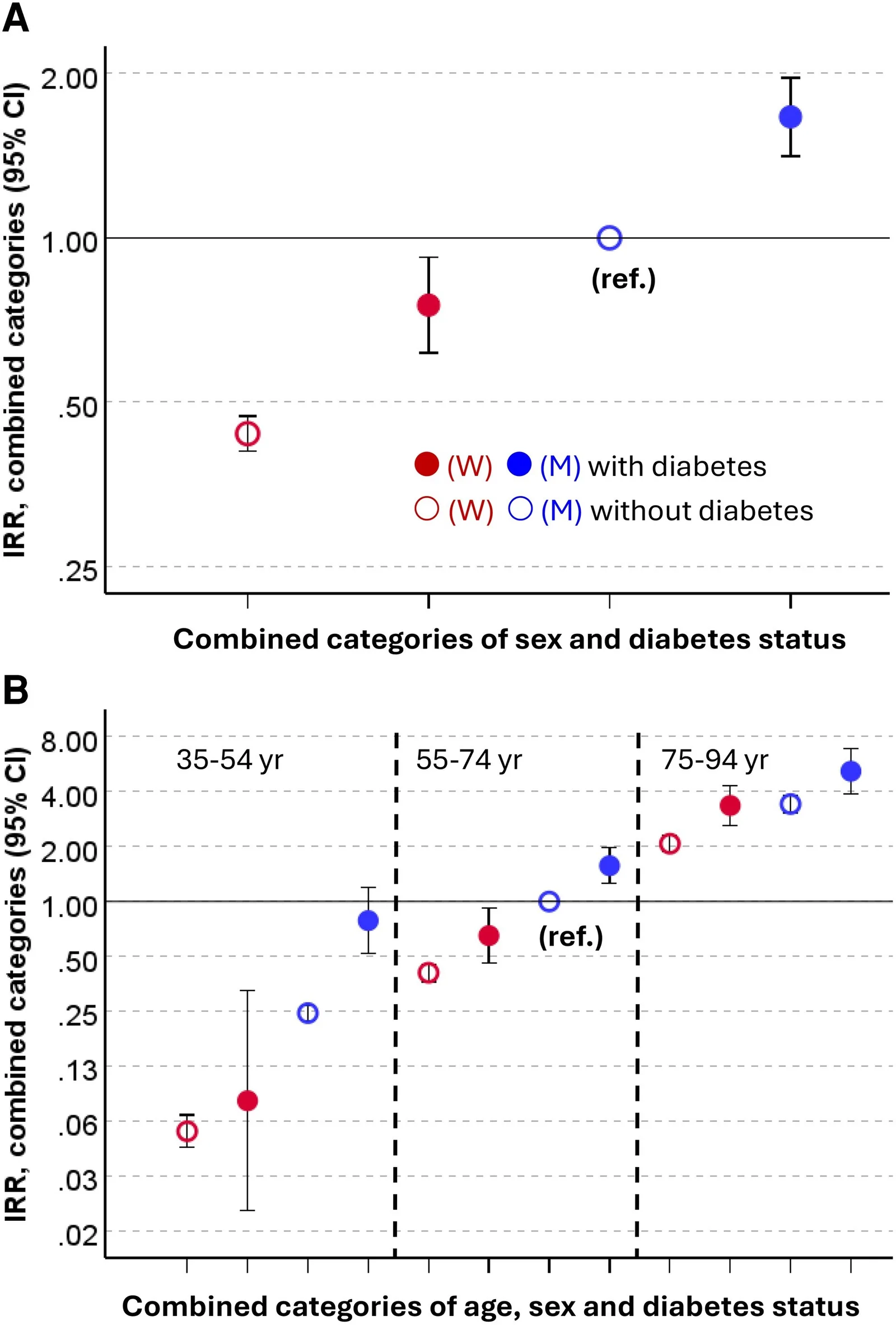
Incidence rate ratio (IRR with 95% confidence interval, CI) of myocardial infarction in subgroups defined by (*A*) sex and diabetes status (men without diabetes as common reference group) and (*B*) sex, age group and diabetes status (men aged 55–74 years without diabetes as common reference group). The IRRs are mutually adjusted for the triglyceride-to-high-density-lipoprotein cholesterol ratio, systolic blood pressure, body mass index and smoking status. W, women; M, men.

### CHD risk factors levels before and after diabetes onset

Temporal changes in the triglyceride-to-HDL-C ratio, systolic blood pressure and BMI (used as adjustment factors in Model 1, *Table 3*) are shown in *Figure 3*. In both sexes, the triglyceride-to-HDL-C ratio (*Figure 3A*), as well as the levels of each of these two lipid variables (see Supplementary material online, *Figure S4A* and *B*), gradually worsened in the years before diabetes onset but improved or stabilized shortly before and after diabetes onset. Individuals with a subsequent incident MI after being diagnosed with diabetes had on average more unfavourable lipid levels, especially after diabetes onset. This contrast was due both to a more pronounced worsening in the prediabetic phase and a less clear improvement after diabetes onset (*Figure 3A*, Supplementary material online, *Figure S4A* and *B*).

**Figure 3:**
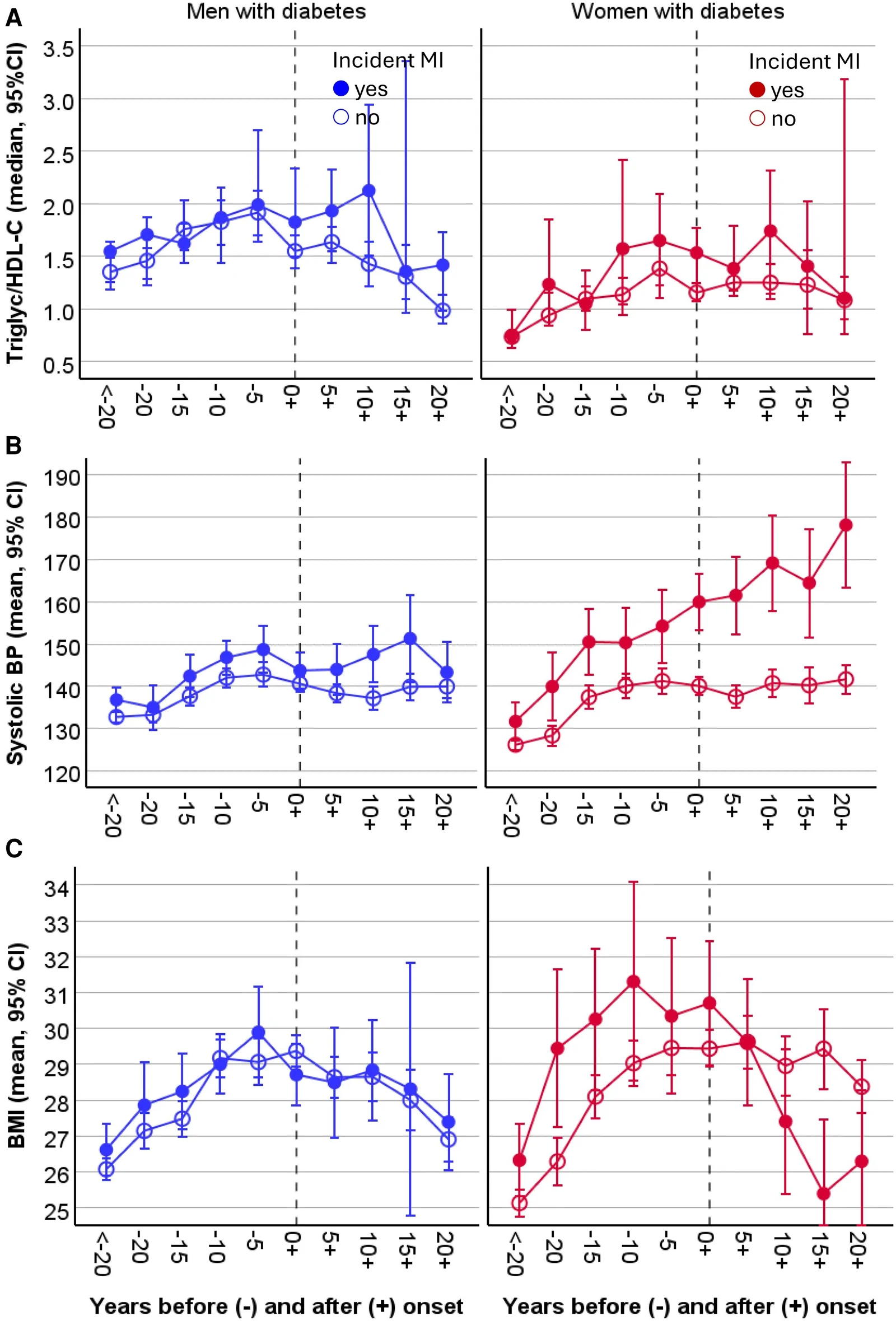
Sample means or median (with 95% confidence interval, CI) of (*A*) the triglyceride to HDL-C ratio, (*B*) systolic blood pressure (mmHg), and (*C*) body mass index (BMI), before and after diabetes onset (5-year groups) in men and women with and without a subsequent incident myocardial infarction (MI).

Systolic blood pressure increased in the years before diabetes onset and continued to rise after diabetes onset, though only in women with a subsequent incident MI (*Figure 3B*). The difference according to MI status in women was substantial and increased with increasing duration of diabetes, whereas the corresponding difference in men was less pronounced and not notably affected by diabetes onset (*Figure 3B*).

In both sexes, BMI increased steadily in the years before diabetes onset, reaching obesity levels 5–10 years prior to diabetes onset, but decreased thereafter (*Figure 3C*). BMI was on average considerably higher in women with than without a subsequent incident MI before diabetes onset but lower afterwards (*Figure 3C*). No clear contrast in mean BMI according to MI status at the end of follow-up was seen among men, neither before nor after diabetes onset (*Figure 3C*).

### Analyses accounting for potential diagnostic delay in diabetes onset

Adjusted for age and birth cohort, the estimated IRR in the analysis with a 10-year backdating of diabetes onset (see Supplementary material online, *Table S3*) was similar to the original value in women (IRR = 2.24, 95% CI = 1.86–2.69) but lower in men (IRR = 1.75, 95% CI = 1.51–2.02). This sex difference reached statistical significance (*P* = 0.040, test for interaction). In men, adjustment for the triglyceride-to-HDL-C ratio had a larger impact than in the original analyses (−20.8% vs. −17.1%). The joint impact of all adjustment factors (applying Model 1) was also larger (−26.3% vs. −15.9%), approaching the almost unchanged impact in women (−33.8%). In the fully adjusted analysis, the adverse effect of diabetes was slightly larger in women (IRR = 1.70, 95% CI = 1.41–2.06) than in men (IRR = 1.51, 95% CI = 1.30–1.75), but no longer statistically significant (*P* = 0.31, test for interaction).

## Discussion

Results from the present large population-based study indicate that traditional CHD risk factors contribute substantially to the diabetes-related elevated risk of MI. Diabetic dyslipidaemia in terms of high triglyceride levels in combination with low HDL-C levels contributed the most to the elevated risk, with similar impact in men and women. Elevated systolic blood pressure contributed markedly in women but not in men. With a larger joint impact of adjustment factors in women, the remaining unexplained diabetes-related elevated risk of MI did not differ notably by sex. Before the age of 55 years, however, an adverse effect of diabetes was seen only in men.

Most previous studies^1–5^ have reported a stronger adverse effect of diabetes on the risk of MI in women than in men, but the relative risk estimates have differed. The present results are consistent with previous findings of a lack of sex difference after adjustment for established CHD risk factors,^23^ although the estimated strength of association varied according to the model applied. In our analyses accounting for a stronger association between systolic blood pressure and the risk of MI in women, the relative risk estimates even indicated a stronger adverse effect of diabetes in men. The choice and modelling of adjustment variables when examining cardiac consequences of diabetes thus needs to be considered carefully and in view of biological relevance, potential intercorrelation between variables as well as aim of the study. The present finding of a considerably weaker impact of triglyceride and HDL-C in a mutually adjusted analysis indicated collinearity problems, making it difficult to adjust for both factors simultaneously. However, adjustment for the triglyceride–to-HDL-C ratio had the largest impact of all CHD risk factors considered. Diabetic dyslipidaemia is characterized by an imbalance in the levels of these lipid variables, and the ratio between them is thus biologically interpretable. The lack of impact of BMI in the mutually adjusted analyses is consistent with recent findings of an intercorrelation between BMI and the lipid variables involved in diabetic dyslipidaemia, especially triglyceride level.^24^

Adjustment for smoking strengthened rather than weakened the association between diabetes and the risk of MI in the present study, especially in men. However, individuals with diabetes are strongly encouraged to quit smoking, and the lower proportion of smokers among individuals with diabetes will thus lead to underestimation in analyses without adjustment for smoking habits. Adjustment for total cholesterol also strengthened the association with diabetes, but to a less extent than the weakening impact of the triglyceride-to-HDL ratio. The large variation in previously reported relative risk estimates for diabetes may relate to what factors were adjusted for. Heterogeneity in the definition of outcome may also play a role.

Consistent with previous reports,^6–10^ we found that women with diabetes have a crude absolute risk of MI close to and even slightly higher than the risk in men without diabetes, though only from ages around 55 years in our study. The similarity in risk between women with diabetes and men without diabetes has typically been interpreted as if diabetes outweighs the female advantage in risk.^1^ However, diabetes increases the risk also in men, and both in the present and previous studies^6–10^ the risk of MI at any age was highest in men with diabetes. In accordance with previous findings^6,8–10,25^ the relative risk estimates for diabetes declined with increasing age, though only in men in our study. The stronger adverse effect of diabetes among the youngest men remained in our analyses accounting for the lower absolute risk of MI in young compared to older people. The lack of adverse effect of diabetes before age 55 years in women, as seen in the present and a previous study,^7^ may reflect a longer latency time for MI in women. However, our age-specific risk estimates, especially in the youngest age group, were very imprecise. Other studies^10,24^ have found an adverse effect of diabetes in women also before age 55 years, though less pronounced than in men in one study.^10^ Different age distributions of study populations may contribute to the variation in previously reported overall relative risk estimates.

### Interpretation in view of CHD risk factor levels before and after diabetes onset

In general, it is difficult to distinguish between confounders, mediators and shared risk factors in a mutually adjusted model without additional information about the internal order of changes in risk factor levels. The present finding of a worsening in triglyceride and HDL-C levels in the time periods before diabetes onset points in the direction of shared risk factors for diabetes and MI. However, as noted by others,^26,27^ it is more likely that insulin resistance in the prediabetic stages is the main cause of the dyslipidaemia. In that case, diabetic dyslipidaemia can be considered as a mediator in the adverse effect of diabetes on the risk of MI. The ratio between triglyceride and HDL-C levels has even been suggested as a marker for insulin resistance.^28,29^ The marked improvement in lipid levels after diabetes onset is apparently contradictory, but the improvement was less clear in the subgroup with a subsequent incident MI and the difference according to MI status seemed to increase after diabetes onset.

The sharp increase in systolic blood pressure after diabetes onset in women with a subsequent incident MI suggests a mediating role of this well-known CHD risk factor, though only in women. Overweight and obesity in individuals with diabetes, a pattern more evident in women than men both in the present and previous studies,^13,14^ may contribute to the larger contrast in systolic blood pressure according to diabetes status in women.^5^ However, the marked decrease in BMI after diabetes onset, especially in women with a subsequent incident MI, is in contrast to the pattern seen for systolic blood pressure. Arterial stiffness in individuals with diabetes may also influence blood pressure,^30,31^ but it is less clear whether there are any sex differences in this potential relationship.

A larger proportion of individuals with than without diabetes reported they had quit smoking. Since quitting smoking is an individual choice, we consider smoking status as a potential confounder in analyses of the adverse effect of diabetes. Although results from our explorative analyses of temporal changes in CHD risk factors are informative, it is difficult to determine the exact role of these factors in relation to the diabetes-related elevated risk of MI.

### Interpretations of results in view of hypothesized biological mechanisms

A main finding in the present study was that high triglyceride levels and low HDL-C levels contribute the most to the diabetes-related elevated risk of MI in both men and women. High levels of low-density lipoprotein cholesterol (LDL-C) are considered as the most important risk factor of atherosclerotic cardiovascular disease,^12,32,33^ and there exist theories on how insulin resistance and hypertriglyceridaemia in individuals with diabetes relate to the mechanisms involved in the production/removal of LDL-C.^11,12^ A recent study^10^ found an elevated risk of cardiovascular disease only in diabetic individuals with confirmed atherosclerosis. Another recent study^34^ found a correlation between severity of atherosclerosis and the value of the triglyceride-to-HDL-C ratio, the factor with the largest impact on the diabetes-related elevated risk of MI in the present study. While some have highlighted the adverse impact of elevated triglyceride levels,^12,35^ others have emphasized the importance of HDL-C and HDL-C subtypes for preventing plaque build-up in arteries.^36^ Results from the present study suggest that the levels of both these lipid variables, and especially the ratio between them, are of importance for the diabetes-related elevated risk of MI.

Duration of exposure to adverse lipid levels is assumed to be the key factor for development of atherosclerosis and subsequent increased risk of MI,^32,33^ with diabetes mentioned as a factor that may accelerate this process.^37^ Results from the present and previous studies^1,14,25,26^ indicate that dyslipidaemia in the prediabetic phase contributes to the time window with exposure to adverse lipid levels. Our finding of a larger impact of adjustment for the triglyceride-to-HDL-C ratio in the analyses with a 10-year backdating of diabetes onset in men also supports such a conclusion if one assumes that the 10-year period before reported age at onset covers a prediabetic phase^1^ and not only reflects diagnostic delay. Whether the CHD risk factor levels adjusted for in these analyses reflect prediabetic levels also depends on the assessment of data within this 10-year time frame.

### Potential clinical implications

The worsening in CHD risk factor levels, as well as increasing BMI in the years before diabetes onset, followed by a marked improvement after the onset of diabetes, highlight the need for early management. Increased awareness of elevated blood glucose levels and early detection of insulin resistance possibly affecting lipid metabolism^26,27^ may thus be important for preventing subsequent cardiac events. Intervention at an early stage, even before reaching the predefined clinical limits for being considered as having diabetes, may need to be considered, especially in individuals with a poor response on statin treatment. Surveillance of blood pressure level after diabetes onset appears to be particularly important in women. The present finding of a larger impact of adjustment for CHD risk factors in men in the analyses with a 10-year backdating of diabetes onset, in contrast to findings in women, may indicate that diagnostic delay is a larger problem in men than women.

### Strengths and limitations

A major strength of the present study was the access to repeated measurements of CHD risk factors, making it possible to update risk factor levels during follow-up, minimizing potential misclassification bias. The longitudinal design of the Tromsø Study also made it possible to explore changes in CHD risk factor levels according to diabetes onset, providing valuable supplementary information. Age at the survey participation was used as a substitute if age at diagnosis of diabetes was unknown, possibly leading to inaccuracies in the crude age-incidence curves of MI in the subgroups with diabetes. A potential under-reporting of diabetes would most likely lead to an underestimation of the adverse effect of diabetes. Unknown confounders and competing events may also lead to bias in risk estimates, but it is difficult to evaluate the direction of potential impact. Although adjustment for leisure time physical activity had a minor impact on the adverse effect of diabetes in the present study, more accurate physical activity measurements may have a larger impact. We could not distinguish between type 1 and type 2 diabetes, but our results will be dominated by the great majority of type 2 diabetes.

### Summary and conclusion

Results from the present study indicate that unfavourable CHD risk factor levels contribute substantially to the diabetes-related elevated risk of MI. The impact was even larger in women than in men. The gradual worsening of CHD risk factor levels during the prediabetic phase is noteworthy. We believe results from the present study contribute to increased knowledge regarding the role of traditional CHD risk factors in relation to sex differences in the cardiac consequences of diabetes.

## Supplementary Material

oeag174_Supplementary_Data

## Data Availability

Data from the Tromsø Study are available upon request (https://uit.no/research/tromsoundersokelsen/project?pid=707286&p_document_id=705235) given necessary permissions from Norwegian authorities (https://helsedata.no/no/variabler/?datakilde=K_TR&page=search).

## Notes

**Consent:** Collection and linkage of data was approved by the Norwegian Data Inspectorate and the Regional Committee for Medical and Health Research Ethics. Written informed consent for participating in the Tromsø Study was introduced in 1994 (fourth and later surveys).
