# Supplementary material for: Sex differences in the diabetes-related risk of incident myocardial infarction and relationship to established coronary heart disease risk factors: the Tromsø Study (1979–2016)
Source: Eur Heart J Open. 2026 Oct 21;6(6):oeag174. doi: 10.1093/ehjopen/oeag174 (PMC13728337; doi:10.1093/ehjopen/oeag174)
Supplement: Supplementary file 1 [file oeag174_supplementary_data.pdf]

SUPPLEMENTARY MATERIAL

Sex differences in the diabetes-related risk of incident myocardial infarction and relationship to established CHD risk factors. The Tromsø Study (1979-2016)

Grethe Albrektsen[1], Tom Wilsgaard[2], Ivar Heuch[3], Dag S. Thelle[4,5], Maja-Lisa Løchen[6,7], Sameline Grimsgaard[2], and Kaare H. Bønaa[8,9]

[1] Department of Public Health and Nursing, Faculty of Medicine and Health Sciences, NTNU Norwegian University of Science and Technology, Trondheim, Norway
[2] Department of Community Medicine, Faculty of Health Science, UiT The Arctic University of Norway, Tromsø, Norway
[3] Department of Mathematics, University of Bergen, Norway
[4] Department of Biostatistics, Institute of Basic Medical Sciences, University of Oslo, Norway
[5] School of Public Health and Community Medicine, Sahlgrenska Academy, Gothenburg University, Sweden
[6] Department of Clinical Medicine, UiT The Arctic University of Norway, Tromsø, Norway
[7] Department of Cardiology, University Hospital of North Norway, Tromsø, Norway
[8] Heart and Thorax Clinic, St. Olavs University Hospital, Trondheim, Norway
[9] Department of Circulation and Medical Imaging, Faculty of Medicine and Health Sciences, NTNU Norwegian University of Science and Technology, Trondheim, Norway

CONTENT:

Statistical Analyses: Details on the use of time-dependent variables and construction of the person-year at risk table used as input data file in the Poisson regression analyses.

Supplementary Figure S1-S3: CHD risk factors levels by age at measurement (5-year groups) in men and women with and without diabetes. The Tromsø Study 1979-2016.

These figures illustrate the difference in mean (or median) lipid and blood pressure variables and BMI between individuals with and without diabetes during adult lifetime, and sex differences in this contrast, based on data from the Tromsø Study 1979-2016.

Supplementary Figure S4: Lipid levels before and after diabetes onset (5-years groups) in men and women with and without a subsequent incident myocardial infarction. The Tromsø Study 1979-2016.

These figures illustrate changes in triglyceride and high-density lipoprotein cholesterol levels in time periods before to after diabetes onset in men and women, and differences in the time patterns and mean levels between individuals with and without a later heart attack, based on data from the Tromsø Study 1979-2016.

Supplementary Table S1: Relative risk estimates (IRR, diabetes vs. non-diabetes) of incident myocardial infarction estimated in Poisson regression analyses of person-years at risk. Impact of adjustment for CHD risk factors.

Supplementary Table S2: Age-specific relative risk estimates (IRR, diabetes vs. non-diabetes) and impact of adjustment for CHD risk factors in each age group.

Supplementary Table S3: Estimated relative risk (IRR, diabetes vs. non-diabetes) of incident myocardial infarction, with a 10-year backdating of diabetes onset to account for a potential diagnostic delay. Impact of adjustment for CHD risk factors.

Supplementary information, Statistical analyses

Diabetes status as time-dependent variable in calculation of crude IR
The time (i.e. age)-dependent indicator variable for diabetes was defined according to reported age at onset of diabetes when calculating the crude age incidence rate (IR) of myocardial infarction (MI). Thus, individuals reporting having diabetes during follow-up contributed with person-years at risk in the non-diabetes group until age at diabetes onset, and in the diabetes group thereafter. Individuals who had diabetes at their first survey participation was considered at risk in the diabetes group until defined end of follow-up (date of MI, emigration, death or closing date of study). Individuals who had diabetes at date of diagnosis of MI (recorded in the Norwegian Myocardial Infarction Register) but not at their last health survey participation before diagnosis of MI, contributed person-years at risk in the diabetes group the last year before diagnosis of MI (to limit misclassification bias in the calculation of crude incidence rate of MI).

Generation of the person-year at risk table used in the Poisson regression analyses
In the generation of the person-years at risk table with number of MI cases and person-years at risk in different risk categories during follow-up (data on aggregated group level), age (in 1-year intervals) was defined as time scale, sex and birth cohort were time-independent variables, whereas all other risk factors considered were treated as time (i.e. age)-dependent variables, allowing for a shift in risk group during follow-up. An individual thus contributed with person-years at risk according to risk factor measurements from the most recent survey participation during follow-up. The time-dependent indicator variable for diabetes was defined according to status at age of survey participation, corresponding in time to CHD risk factor measurements. The Poisson regression analyses were restricted to the age interval 35-94 years, and the risk factor levels at start of follow-up were in these analyses defined according to measurement closest in time before the age of 35 years (for individuals with first survey participation at ages 20-35 years) or to risk factor levels measured at first survey participation at a higher age (delayed entry).

When constructing the person-years at risk table with data on aggregated group level, blood pressure and lipid variables were categorized into small, equal-sized, clinically relevant intervals. Systolic blood pressure was categorized into eight 10 mmHg intervals, diastolic blood pressure into six 5 mmHg intervals, whereas 5 categories were defined for pulse pressure (<40, 40-49, 50-59, 60-69, ≥70 mmHg). Despite the need for categorization into narrow intervals when generating the person-years at risk table, diastolic and systolic blood pressure were treated as continuous covariates in the Poisson regression model (modelled as linear trend trough ordered categories), whereas pulse pressure was included as a categorical variable due to deviation from a log-linear relation with the risk of MI. Total cholesterol (ten 0.5 mmol/L intervals), high-density lipoprotein cholesterol (HDL-C, six 0.3 mmol/L intervals), triglycerides (eight 0.5 mmol/L intervals), the HDL-C to total cholesterol ratio, recalculated into percentage HDL-C (six 5% intervals) and the triglyceride to HDL-C ratio (seven 0.5 units intervals, broader intervals at highest levels) were also modelled as linear trends through ordered categories in the Poisson regression model. BMI category (underweight, normalweight, overweight, obese I, obese II), daily smoking (no, previous, yes) and self-reported physical activity (low, moderate, high, very high) were kept as categorical covariates in the regression model (some categories combined due to low numbers or similar risk, as described in main text).

Supplementary Figure S1 – CHD risk factor levels at start and during follow-up

Sample means or median (with 95% confidence interval, CI) of (A) total cholesterol (mmol/L), (B) high-density lipoprotein cholesterol (HDL-C, mmol/L) and (C) triglyceride level (mmol/L) by age at measurement (5-year groups) in men and women with (●) and without (○) diabetes.

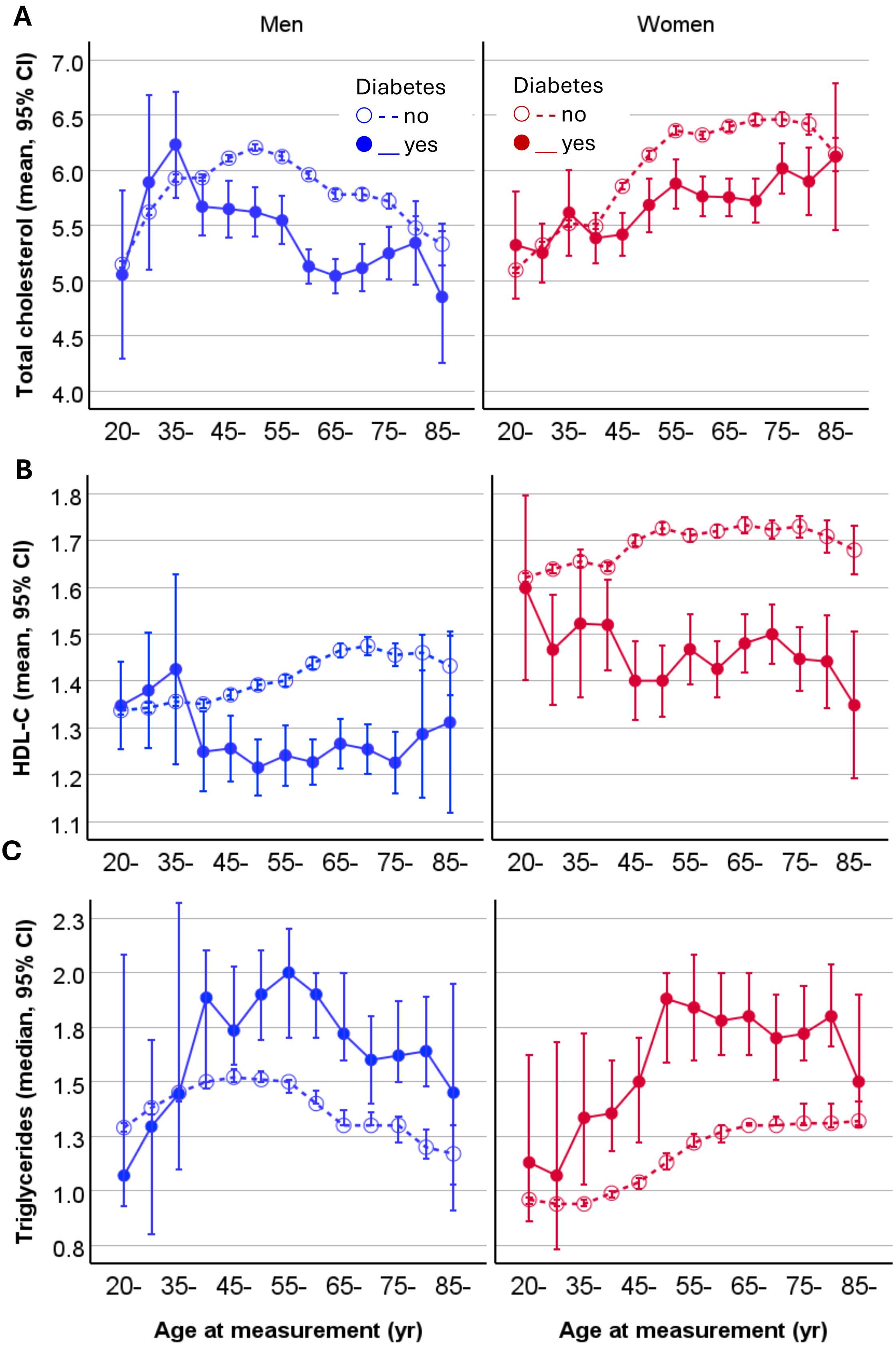

Supplementary Figure S2 – CHD risk factor levels at start and during follow-up

Sample means or median (with 95% confidence interval, CI) of (A) the high-density lipoprotein to total cholesterol ratio (HDL-C/TC, recalculated into %), B) the triglyceride to HDL-C ratio and (C) body mass index, by age at measurement (5-year groups) in men and women with (●) and without (○) diabetes.

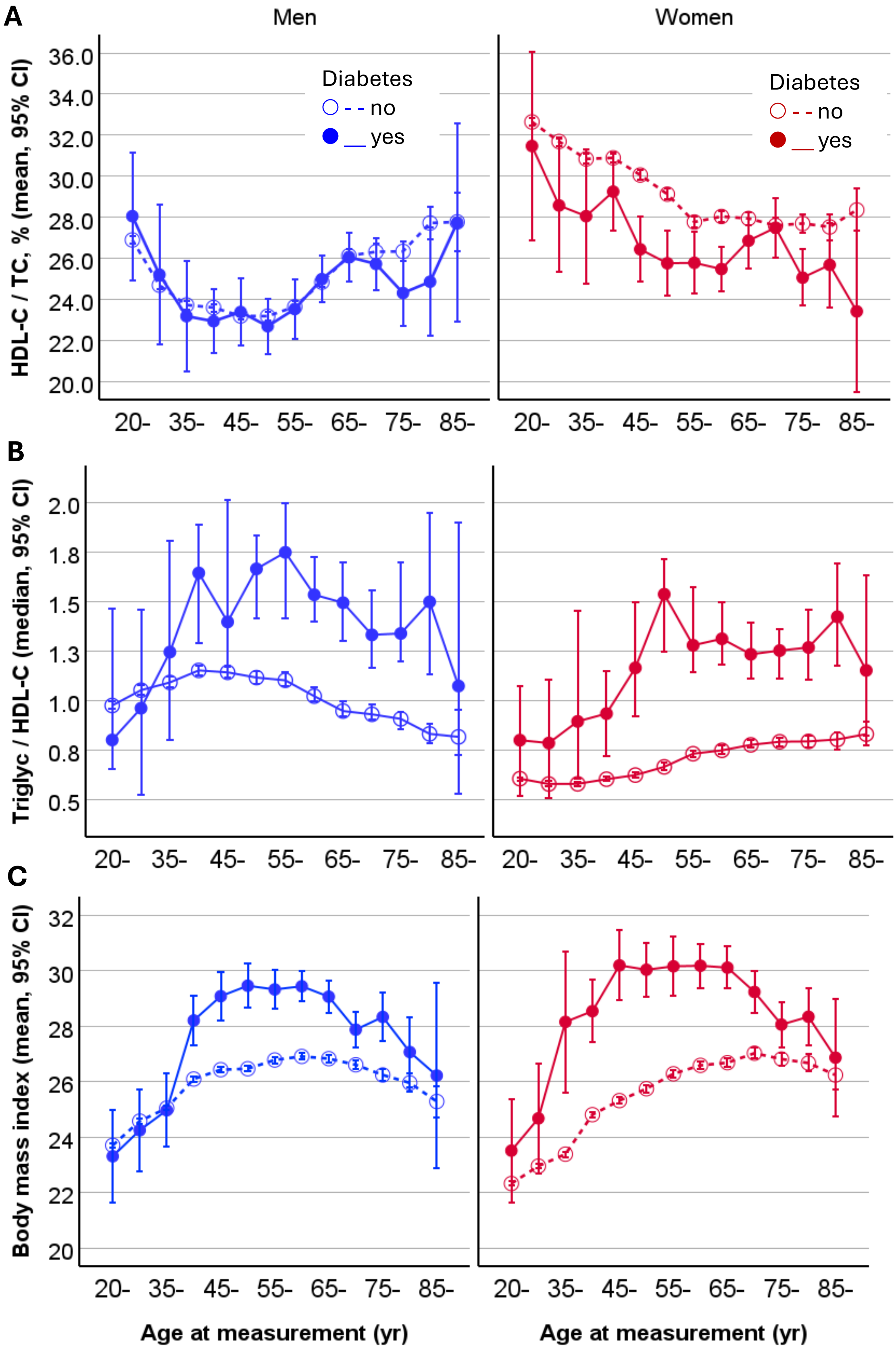

Supplementary Figure S3 – CHD risk factor levels at start and during follow-up

Sample means (with 95% confidence interval, CI) of (A) systolic and (B) diastolic blood pressure (BP, in mmHg) and (C) pulse pressure (systolic BP – diastolic BP) by age at measurements (5-year groups) in men and women with (●) and without (○) diabetes.

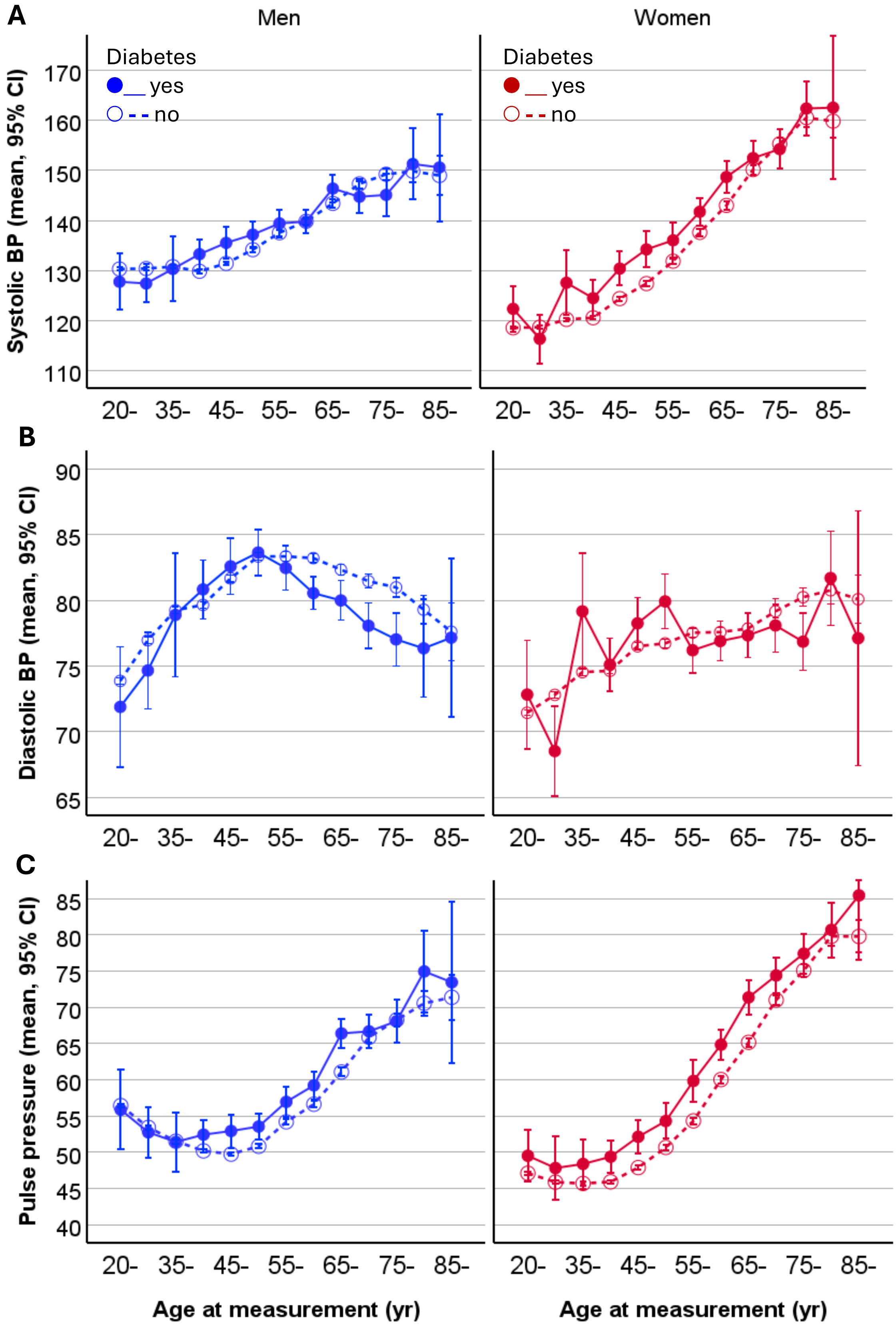

Supplementary Figure S4 – CHD risk factor levels before and after diabetes onset

Sample means or median (with 95% confidence interval, CI) of (A) triglycerides (mmol/L) and (B) high-density lipoprotein cholesterol (HDL-C, mmol/L), before and after diabetes onset (5-year groups) in men and women with (●) and without (○) a subsequent incident myocardial infarction (MI).

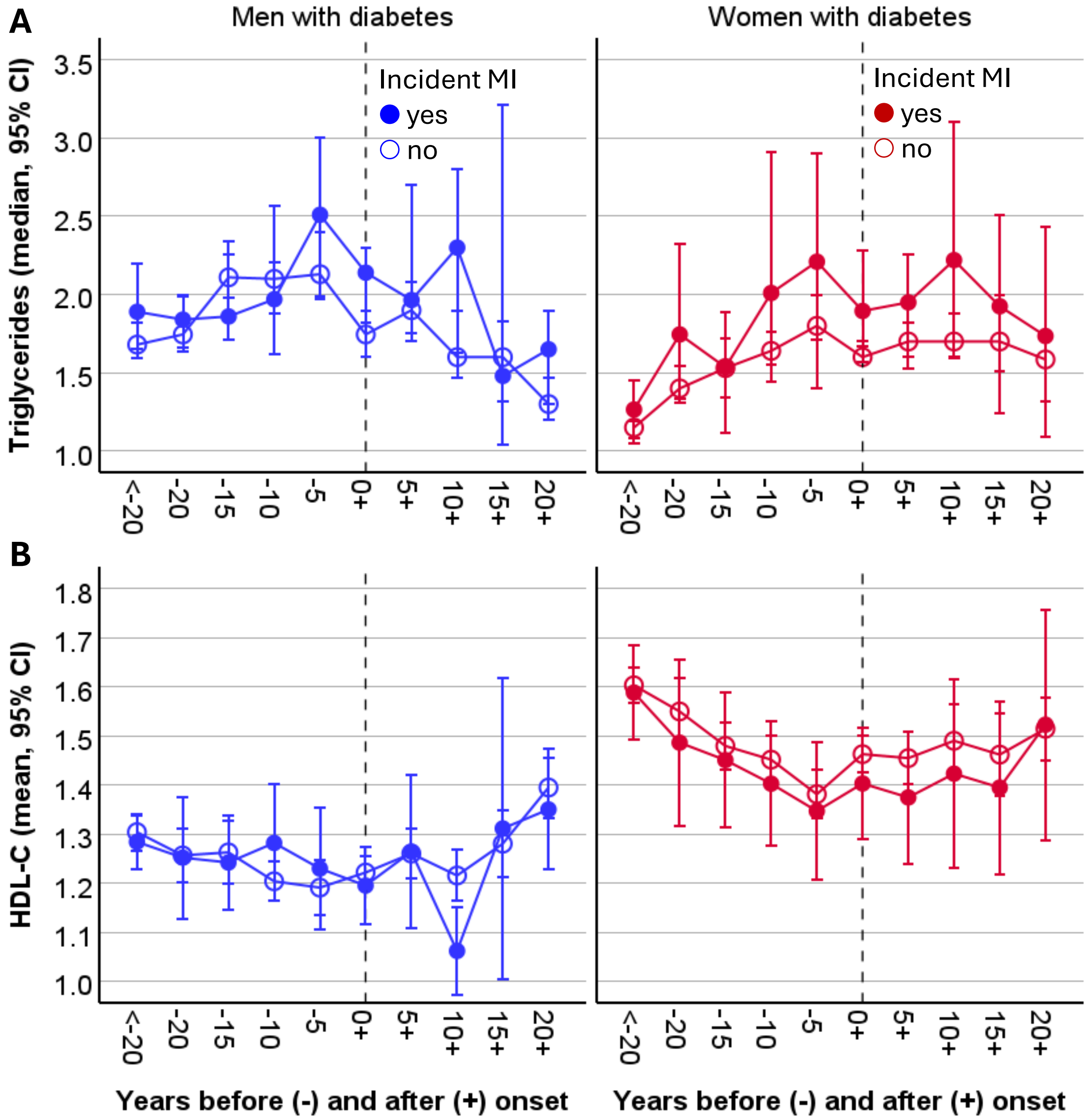

Supplementary Table S1 – Impact of CHD risk factors

Incidence rate ratio (IRR with 95% CI) of myocardial infarction in individuals with vs. without diabetes. Impact of adjustment for CHD risk factors (mutually adjusted model).

	**Men IRR (95% CI)**	**Women IRR (95% CI)**	**Men** Δ%	**Women** Δ%
Adjusted age and birth cohort	**1.84 (1.56-2.16)**	**2.26 (1.85-2.77)**	***Ref.***	***Ref.***
**Additional adjustment**				
**MODEL 3**				
Joint impact, all factors	**1.73 (1.46-2.04)**	**1.79 (1.46-2.20)**	**-10.0**	**-28.5**
**Impact, each single factor**				
Triglycerides	-11.3 %	-12.1 %		
Systolic BP	+2.2 %	-9.2 %		
Body mass index	-4.2 %	-6.3 %		
Daily smoking	+21.0 %	+5.8 %		
**Additional interaction**				
with Systolic BP	**1.77 (1.50-2.09)**	**1.70 (1.38-2.08)**		
	+5.0 %	-9.4 %		
**MODEL 4**				
Joint impact, all factors	**1.73 (1.46-2.04)**	**1.76 (1.43-2.16)**	**-9.9**	**-30.9**
**Impact, each single factor**				
HDL-C	-11.2 %	-15.7 %		
Systolic BP	+0.3 %	-11.7 %		
Body mass index	-4.5 %	-6.2 %		
Daily smoking	+20.1 %	+6.1 %		
**Additional interaction**				
with Systolic BP	**1.78 (1.51-2.10)**	**1.66 (1.35-2.03)**		
	+5.0 %	-10.4 %		

Results from Poisson regression analyses of person-years at risk. The sex-specific IRR values are calculated on basis of a model including an interaction between sex and diabetes status. The joint impact of CHD risk factors is quantified as percentage change (Δ%) in the regression coefficient for diabetes when including all factors simultaneously in a model adjusted for age and birth cohort (reference model). The impact of a single CHD risk factor in a mutually adjusted model is calculated as percentage change when adding this factor in the last step. The impact of including an additional interaction term to allow for sex differences in the effect of systolic blood pressure ($p<0.001$, test for interaction) is also shown. CHD, coronary heart disease; CI, confidence interval; HDL-C, high-density lipoprotein cholesterol; BP, blood pressure.

Supplementary Table S2 – Results from age-specific analyses

Age-specific incidence rate ratio (IRR with 95% confidence interval, CI) of myocardial infarction in individuals with vs. without diabetes. Impact of adjustment for CHD risk factors in a mutually adjusted model (applying Model 1 in main manuscript).

	**Age 35-54 yr**		**Age 55-74 yr**		**Age 75-94 yr**	
	IRR (95% CI)	Δ%	IRR (95% CI)	Δ%	IRR (95% CI)	Δ%
**MEN**						
Adjusted age only	**3.34 (2.20-5.07)**	***Ref.***	**1.83 (1.46-2.30)**	***Ref.***	**1.57 (1.17-2.09)**	***Ref.***
**Additional adjustment**						
Joint impact, all factors	**2.73 (1.79-4.16)**	**-16.7**	**1.65 (1.31-2.07)**	**-17.4**	**1.51 (1.13-2.02)**	***-7.9***
**Impact, each factor**						
Triglyceride/HDL-C	-8.0 %		-16.4 %		-17.7 %	
Systolic BP	-1.7 %		-0.8 %		+4.7 %	
Body mass index	-7.0 %		-5.6 %		-2.9 %	
Daily smoking	+13.9 %		+28.6 %		+15.3 %	
**WOMEN**						
Adjusted age only	**1.85 (1.46-7.49)**	***Ref.***	**2.37 (1.67-3.26)**	***Ref.***	**1.87 (1.17-2.09)**	***Ref.***
**Additional adjustment**						
Joint impact, all factors	**1.13 (0.28-4.58)**	**-80.6**	**1.64 (1.17-2.31)**	**-41.7**	**1.69 (1.31-2.19)**	**-16.0**
**Impact, each factor**						
Triglyceride/HDL-C			-19.1 %		-14.4 %	
Systolic BP			-17.0 %		-5.0 %	
Body mass index			-8.5 %		+2.3 %	
Daily smoking			+1.3 %		+4.8 %	

Results from Poisson regression analyses of person-years at risk within each 20-year age group. The sex-specific IRR values are calculated on basis of a model including an interaction between sex and diabetes status. The joint impact of CHD risk factors is quantified as percentage change (Δ%) in the regression coefficient for diabetes when including all factors simultaneously in a model adjusted for age and birth cohort (reference model). The impact of a single CHD risk factor in a mutually adjusted model is calculated as percentage change when adding this factor in the last step. CHD, coronary heart disease; CI, confidence interval; HDL-C, high-density lipoprotein cholesterol; BP, blood pressure.

Supplementary Table S3 – Results from analyses with backdating of diabetes onset

Incidence rate ratio (IRR with 95% confidence interval, CI) of myocardial infarction in individuals with vs. without diabetes, with a 10-year backdating of diabetes onset to account for a potential diagnostic delay. Impact of adjustment for CHD risk factors in a mutually adjusted model (applying Model 1 in main manuscript).

	**Men** IRR (95% CI)	**Women** IRR (95% CI)	**Men** Δ%	**Women** Δ%
Adjusted age and birth cohort	**1.75 (1.51-2.02)**	**2.24 (1.86-2.69)**		
**Additional adjustment**				
Joint impact, all factors	**1.51 (1.30-1.75)**	**1.70 (1.41-2.06)**	***-26.3***	***-33.8***
**Impact, each single factor**				
Triglyceride/HDL-C (ratio)	-20.8 %	-18.9 %		
Systolic BP	-1.7 %	-10.9 %		
BMI category	-5.6 %	-4.9 %		
Daily smoking (no, yes)	+22.7 %	+9.1 %		
**Additional interaction term**				
with Systolic BP	**1.55 (1.34-1.79)**	**1.62 (1.34-1.96)**		
	+6.3 %	-9.3 %		

Results from Poisson regression analyses of person-years at risk. The sex-specific IRR values are calculated on basis of a model including an interaction between sex and diabetes status. The joint impact of CHD risk factors is quantified as percentage change (Δ%) in the regression coefficient for diabetes when including all factors simultaneously in a model adjusted for age and birth cohort (reference model). The impact of a single CHD risk factor in a mutually adjusted model is calculated as percentage change when including this factor in the last step. The impact of including an additional interaction term to allow for sex differences in the effect of systolic blood pressure ($p<0.001$, test for interaction) is also shown. CHD, coronary heart disease; BP, blood pressure; HDL-C, high-density lipoprotein cholesterol.
